# Efficacy and safety data of ceftiofur antibiotics against *Streptococcus parauberis* PH0710 infection in starry flounder (*Platichthys stellatus*)

**DOI:** 10.1016/j.dib.2019.103729

**Published:** 2019-03-07

**Authors:** Min Soo Joo, Jee Youn Hwang, Mun-Gyeong Kwon, Jung Soo Seo, Seong Don Hwang, Bo-Yeong Jee, Mu-Chan Kim, Chan-Il Park

**Affiliations:** a Institute of Marine Industry, College of Marine Science, Gyeongsang National University, 455, Tongyeong 650-160, Republic of Korea; b Aquatic Animal Disease Control Center, National Institute of Fisheries Science (NIFS), 216 Gijanghaean-ro, Gijang-eup, Gijang-gun, Busan 46083, Republic of Korea

**Keywords:** Antibiotics, Ceftiofur, Starry flounder, *Streptococcus parauberis* PH0710, Efficacy, Safety

## Abstract

This article provides efficacy and safety data of ceftiofur antibiotics against streptococcal infection in starry flounder. Ceftiofur, which is a veterinary antibiotics, is effective against fishery bacteria. Ceftiofur can be prescribed and sold by veterinarians. However, it is illegal in South Korea for fishery disease managers to prescribe and sell ceftiofur. Therefore, in order to utilize available antibiotics and prevent illegal use of veterinary antibiotics, it is necessary to perform research to determine the recommended effective dose and administration methods of antibiotics for fisheries. In this article, the appropriate concentration and injection method of antibiotics to treat starry flounder infected with *S. parauberis* PH0710 were provided. In addition, histopathological examination results were provided to confirm the effect of antibiotics on the host tissue. Accordingly, these data could be used as basic data for the application of ceftiofur antibiotics in disease management for fisheries.

**Table:** Specifications table

Subject area	Veterinary Science and Veterinary Medicine
More specific subject area	Veterinary Medicine
Type of data	Tables and figures
How data was acquired	Measuring cumulative mortality rate. The kidney and spleen of the dead fish were homogenized, suspended in phosphate buffered saline (PBS), and spread on brain heart infusion agar (BHIA) to confirm the infectivity per gram of tissue. Damaged tissues were observed through an optical microscope after hematoxylin and eosin (H&E) stain.
Data format	Raw, analysed, groups.
Experimental factors	Fish were acclimated for 2 weeks under laboratory conditions. Water temperature, flow rate and photoperiod were measured. Bacteria challenge concentration and antibiotics injection concentration were varied.
Experimental features	The following were determined: Therapeutic effects of various concentrations of antibiotics; therapeutic effects of each antibiotic administration method; and toxicity of antibiotics in host cells.
Data source location	Tongyeong, Republic of Korea
Data accessibility	Data are present in this article only

**Table:** 

**Value of the data** • We established appropriate treatment standards for new antibiotics that can inhibit bacterial strains with resistance to antibiotics that have been used previously. • These data are an important baseline for the application of veterinary antibiotics to fisheries management. • By providing effective treatment concentrations and methods for Streptococcal disease, these data are ready to use for aquaculture sites. • These data allows not only veterinarians but also fishery disease managers to prescribe and sell ceftiofur antibiotics.

## 1 Data

**Fig. 1:**
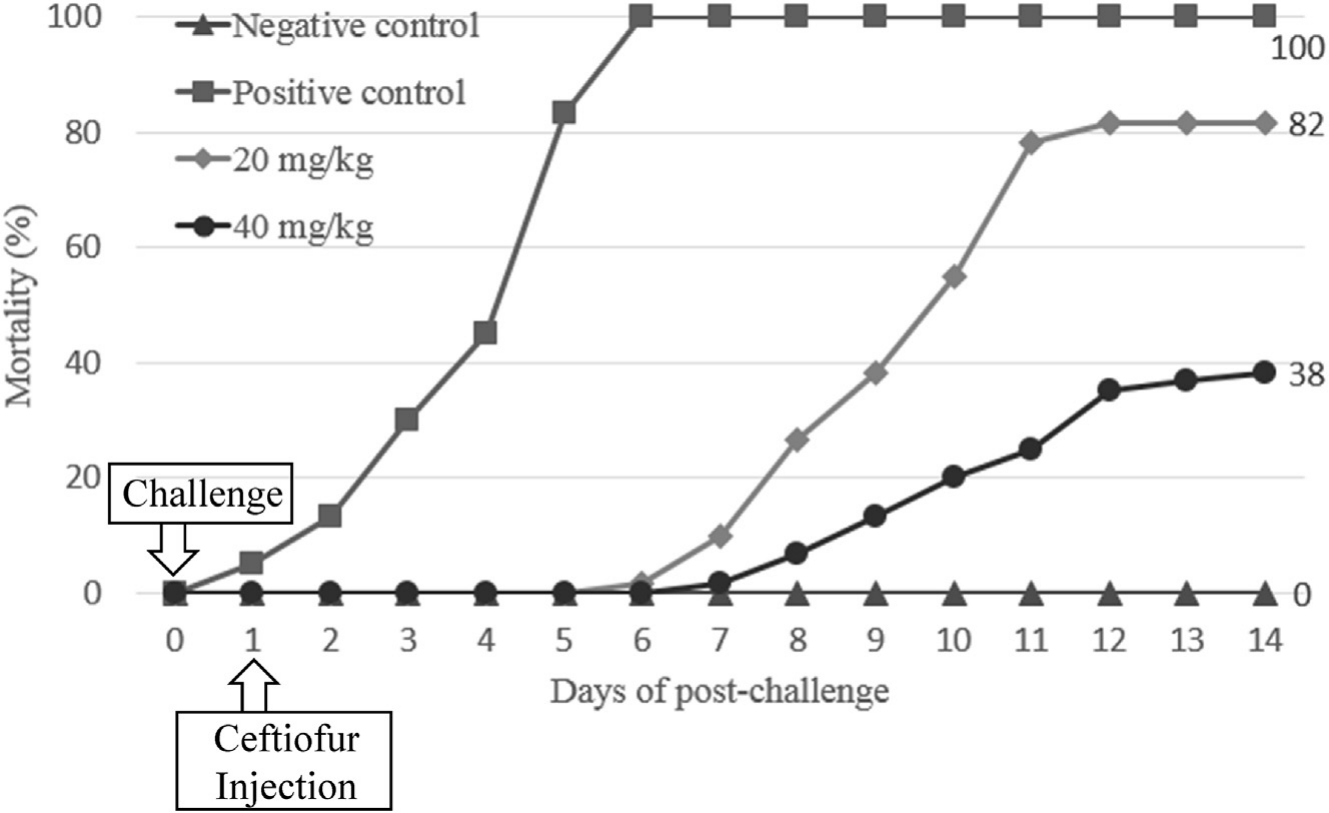
Determination of cumulative mortality from each concentration of ceftiofur against *S. parauberis* PH0710 infection in starry flounder.

**Fig. 2:**
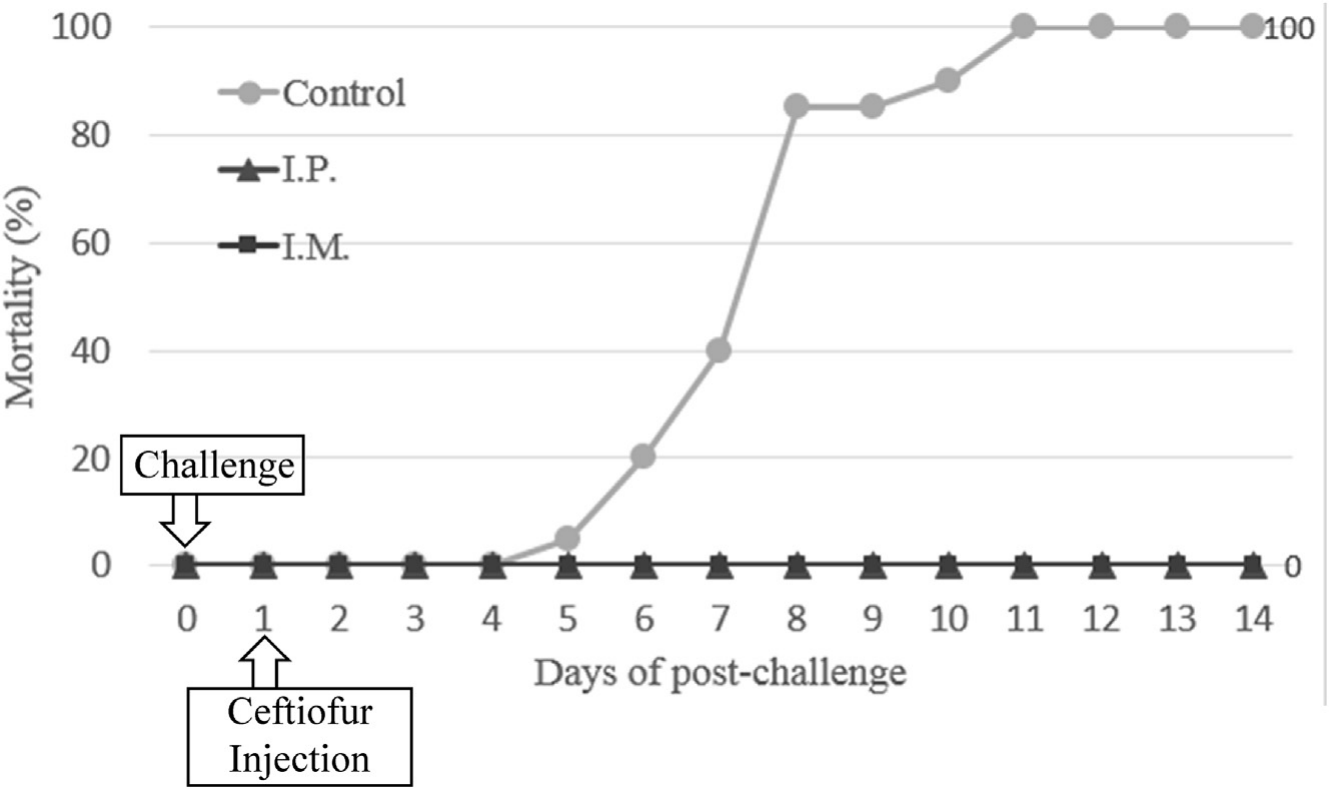
Determination of cumulative mortality from each administration method of ceftiofur against *S. parauberis* PH0710 infection in starry flounder.

**Fig. 3:**
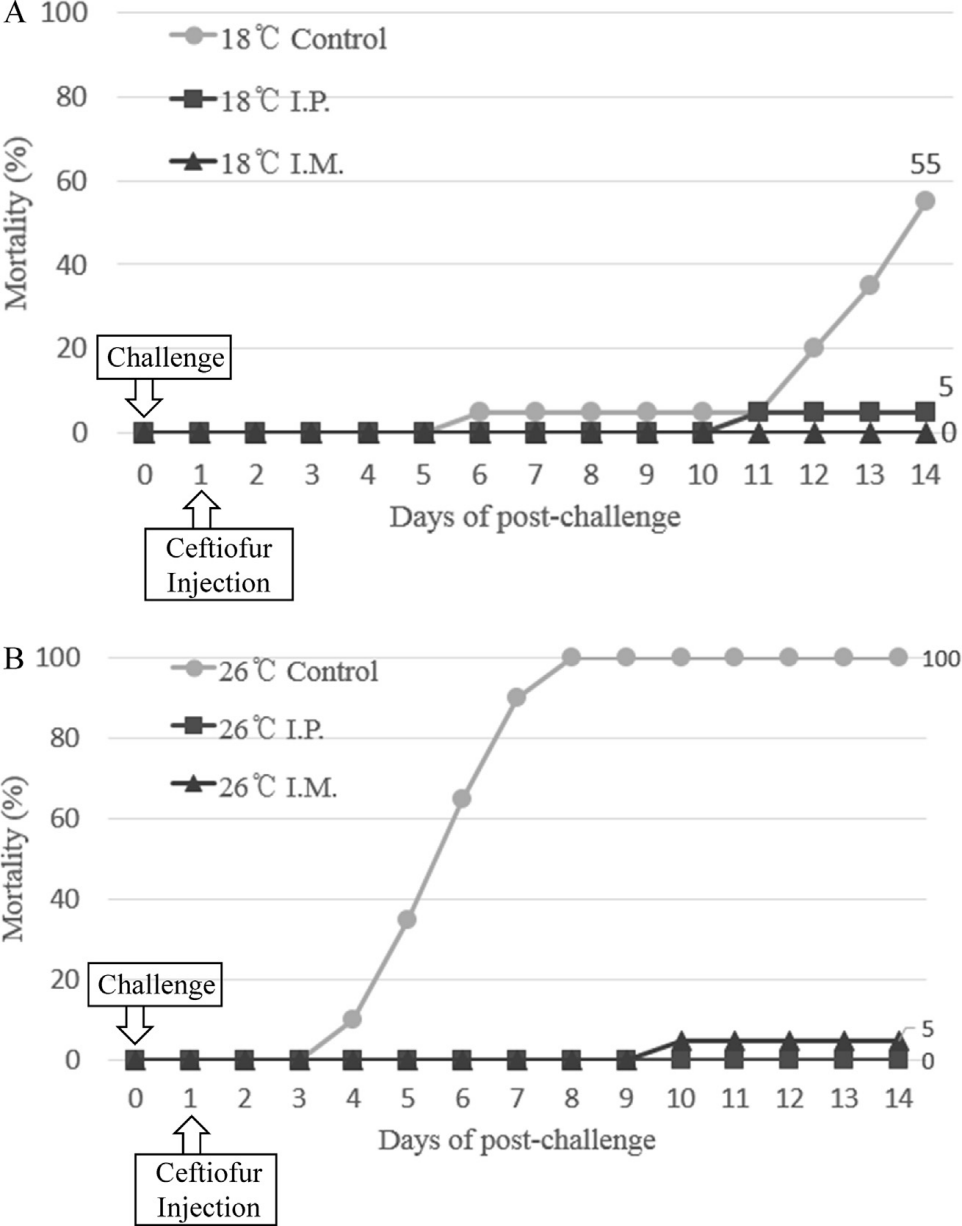
Determination of cumulative mortality from each administration method of ceftiofur against *S. parauberis* PH0710 infection in starry flounder at 18 °C (A) and 26 °C (B).

**Fig. 4:**
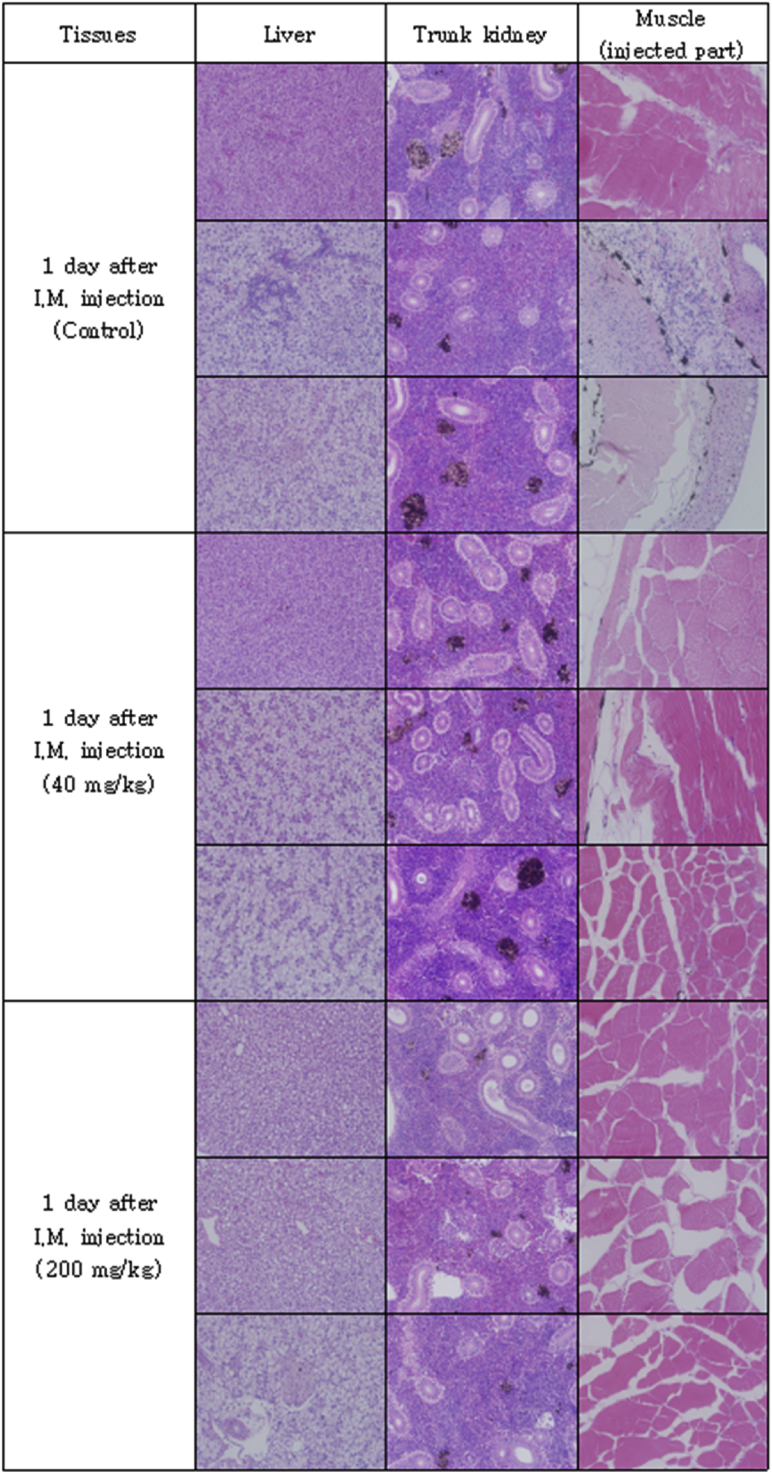

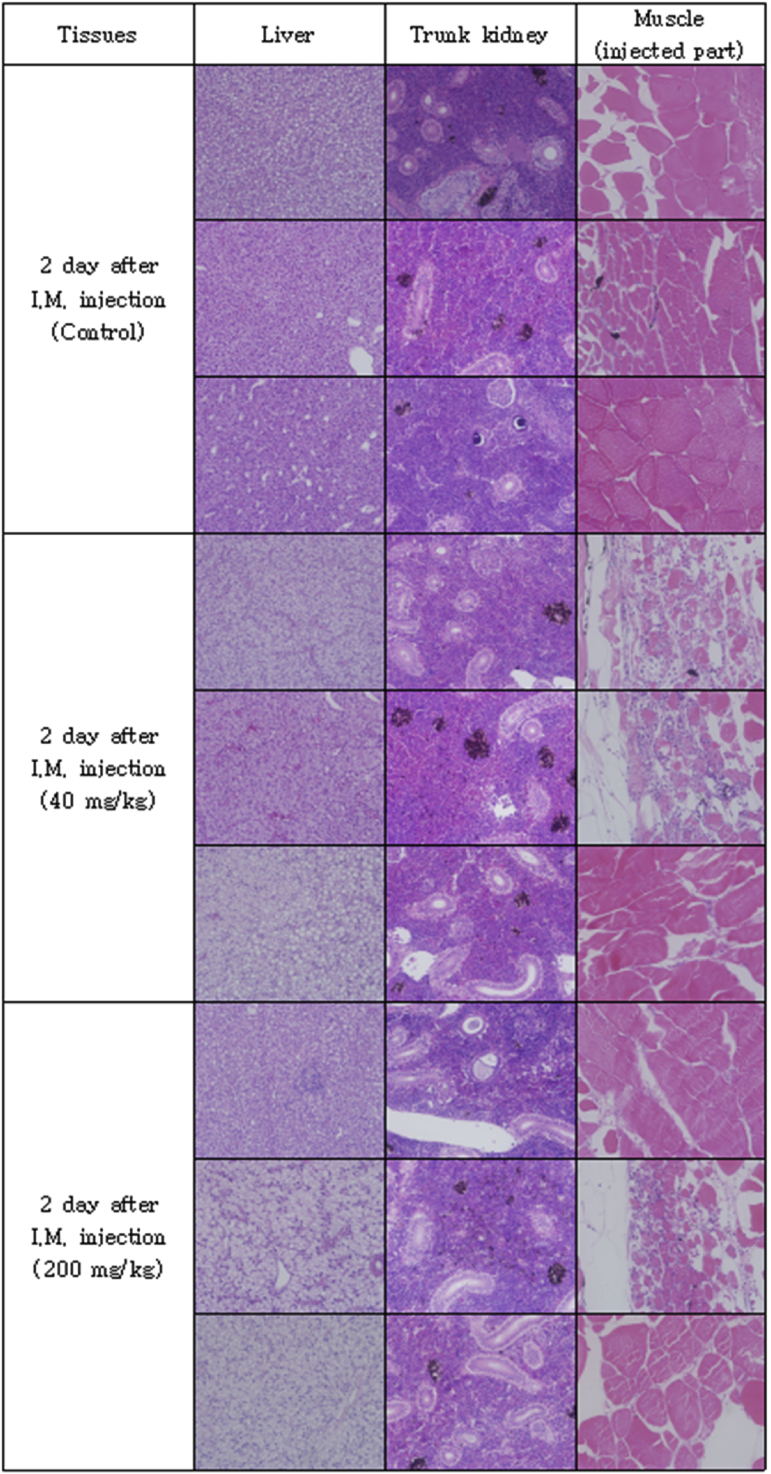

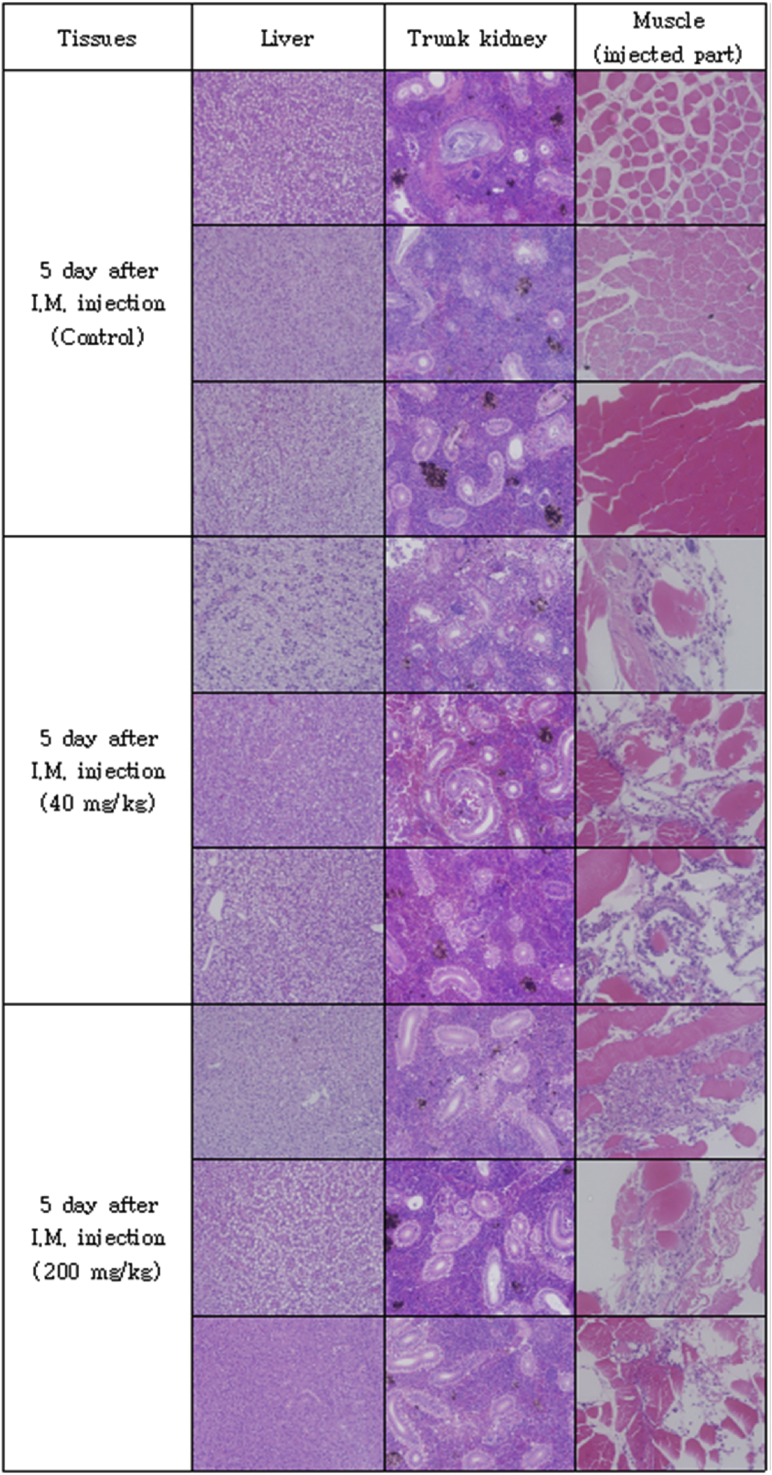

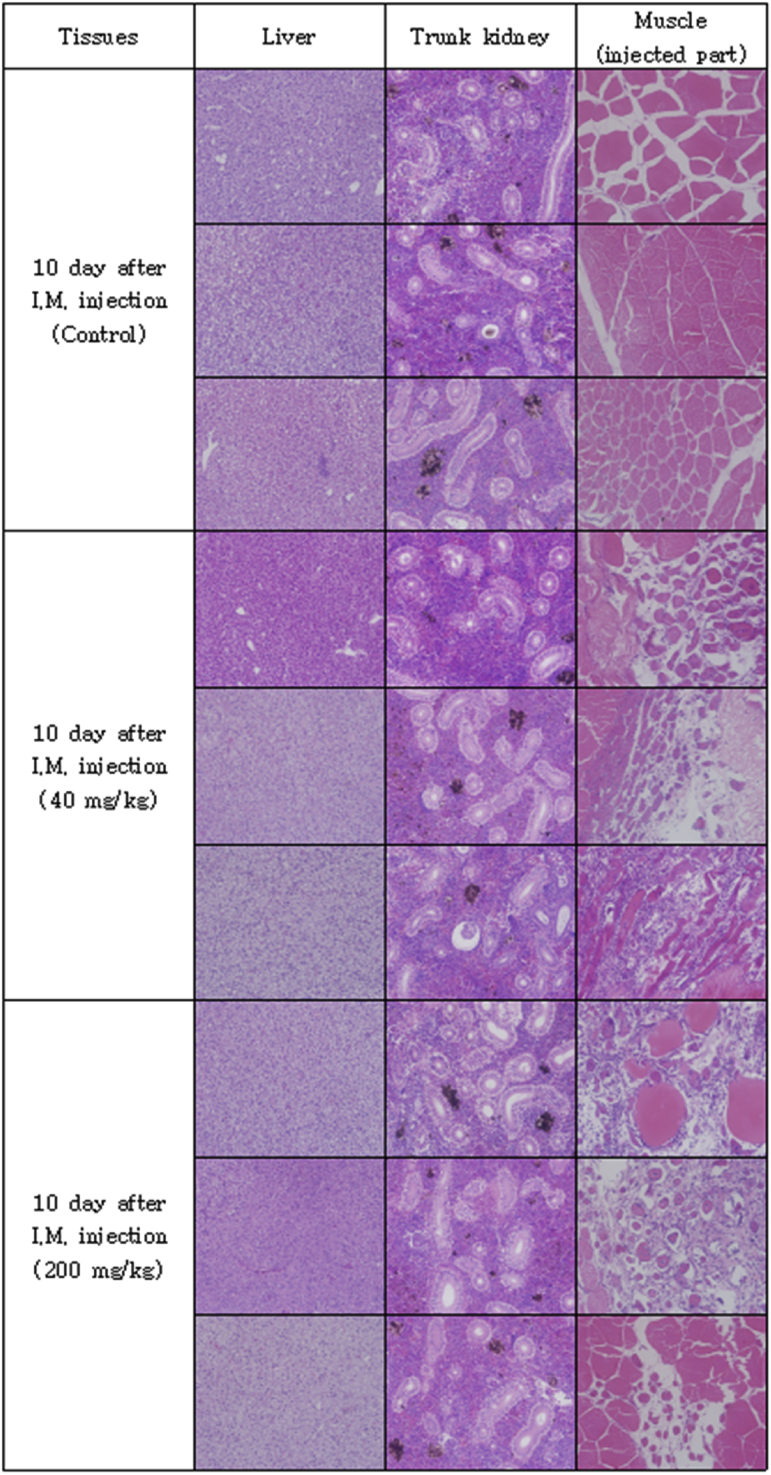

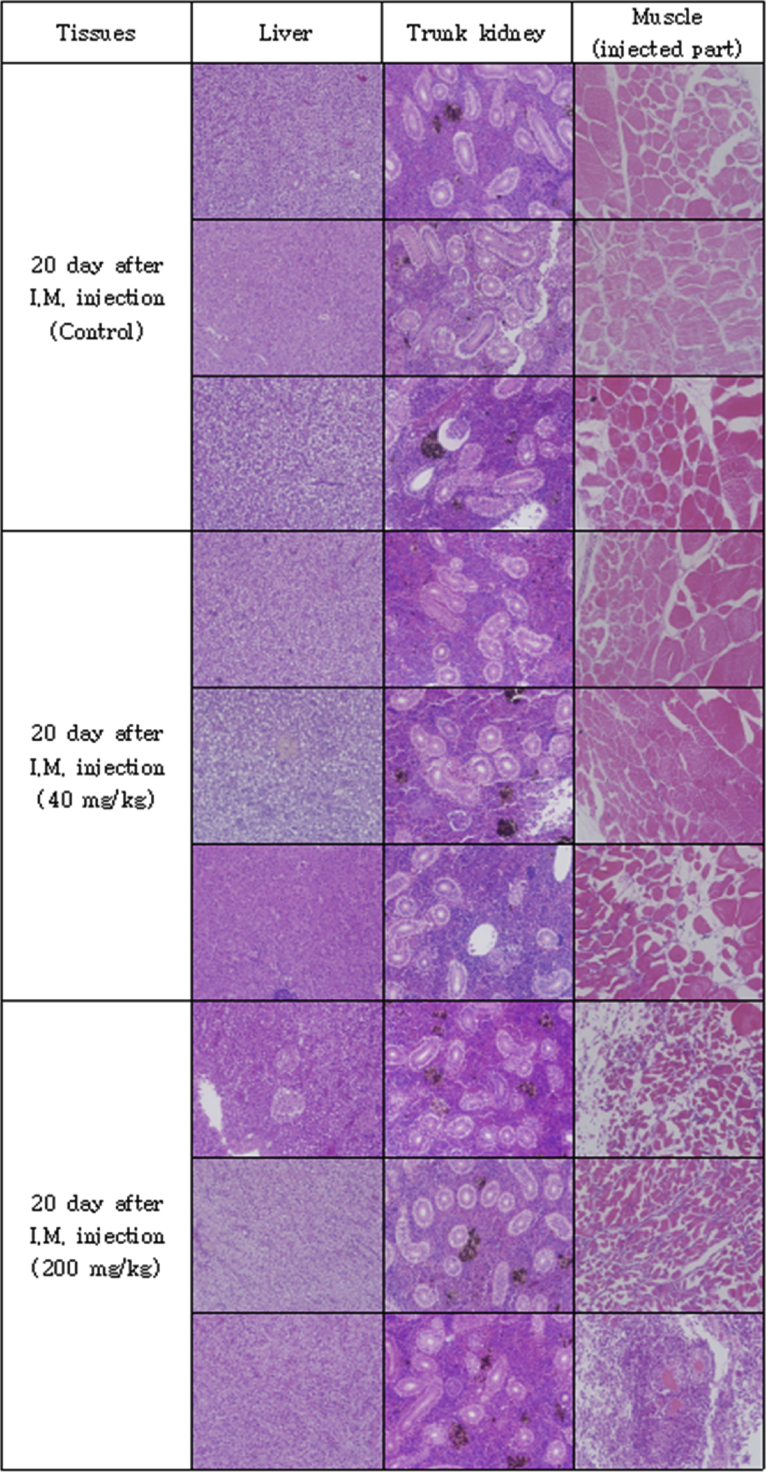
Pathological results of liver, kidney and muscle (injected part) after ceftiofur injection by each concentration.

The optimal concentration of ceftiofur antibiotics for *Streptococcus parauberis* PH0710 infected starry flounder was confirmed (Fig. 1). The cumulative mortality rate, relative survival rate and infection rate in surviving fish were checked for antibiotic efficacy experiments with varied ceftiofur concentrations (Table 1). The efficacy of the antibiotic injection method was not different between the intraperitoneal injection and the intramuscular injection (Fig. 2). The infection rate was not different in surviving fish (Table 2). Experiments at different temperatures showed no significant difference in mortality (Fig. 3), but infection rates were lower in the intraperitoneal injection group than those in the intramuscular injection group (Table 3). Liver atrophy and fatty liver were observed in all fish including the control, and mild glomerulonephritis was common in kidney. There was a significant difference between the control and experimental groups regarding damage of muscle tissue. On days 5, 10 and 20 after injection, an inflammatory reaction was observed in the experimental group, but the control group was normal. On day 20 after injection, severe inflammation was observed in the 200 mg/kg concentration group, but no abnormal symptoms were observed in the 40 mg/kg concentration group (Fig. 4).

**Table 1:** The cumulative mortality, infection rate and relative survival rate of efficacy studies in *S. parauberis* PH0710 infection in starry flounder by ceftiofur dosing concentration.

Group	Con (−)	Con (+)	20 mg/kg	40 mg/kg
Cumulative mortality (%)	0 (0/60)	100 (60/60)	82 (49/60)	38 (23/60)
Relative survival rate (%)	–	–	18	62
Infection rate (%) in survival fish	0	–	36 (4/11)	22 (8/37)

(Corresponding number of fish/Total number of fish).

**Table 2:** The cumulative mortality, infection rate and relative survival rate of efficacy studies in *S. parauberis* PH0710 infection in starry flounder by ceftiofur administration method.

Group	Con (+)	40 mg/kg, I.P.	40 mg/kg, I.M.
Cumulative mortality (%)	100 (20/20)	0 (0/20)	0 (0/20)
Relative survival rate (%)	–	100	100
Infection rate (%) in survival fish	–	0 (0/20)	0 (0/20)

(Corresponding number of fish/Total number of fish).

**Table 3:** The cumulative mortality, infection rate and relative survival rate of efficacy studies in *S. parauberis* PH0710 infection in starry flounder by ceftiofur administration method at each temperature.

Group	18 °C Con(+)	18 °C 40mg/kg, I.P	18 °C 40mg/kg, I.M	26 °C Con(+)	26 °C 40mg/kg, I.P	26 °C 40mg/kg, I.M
Cumulative mortality (%)	55 (11/20)	5 (1/20)	0 (0/20)	100 (20/20)	0 (0/20)	5 (1/20)
Relative survival rate (%)	–	90	100	–	100	95
Infection rate (%) in survival fish	67 (6/9)	5 (1/19)	15 (3/20)	–	5 (1/20)	11 (2/19)

(Corresponding number of fish/Total number of fish).

## 2 Experimental design, materials and methods

### 2.1 Strain

*S. parauberis* PH0710 isolated from starry flounder infected with Streptococcal disease at an aquaculture in Pohang, Gyeongsangbuk-do, South Korea, was used for the experiment. The strain was cultured at 27 °C for 24 hours using brain heart infusion agar (BHIA) supplemented with 1% NaCl. The strains were stored at −80 °C until use.

### 2.2 Identification of the strain

*S. parauberis* PH0710 was identified by polymerase chain reaction (PCR) using specific primers (Table 4). In the subsequent experiments, the bacteria were isolated from the spleen and kidney and confirmed by PCR. At the end of the experiment, the bacteria isolated from the surviving fish were also identified. The conditions for PCR were as follows: 94 °C for 2 min, 25 cycles of 92 °C for 1 min, 55 °C for 1 min, 72 °C for 90 sec, and final extension at 72 °C for 5 min [1].

**Table 4:** Specific primers for *S. parauberis* detection.

Primer	Sequences (5’→3′)	Target gene	Product size (bp)
Spa2152	TTTCGTCTGAGGCAATGTTG	23s rRNA	718
Spa2870	GCTTCATATATCGCTATACT		

### 2.3 Fish

Starry flounder, weighting 51.8 ± 8.56 g, were purchased from an aquaculture in Pohang, Gyeongsangbuk-do, South Korea, with no history of infection with *S. parauberis*. Fish were kept in 250 L running seawater tanks.

### 2.4 Ceftiofur antibiotics

Cefoshot (Korea Thumb Vet, Korea), which consists of ceftiofur sodium 1000 mg per gram of vial contents, was used.

### 2.5 Proper bacterial concentration of artificial infection

To determine the optimal infection concentration of *S. parauberis* PH0710 against starry flounder, infection was performed at various concentrations. The mortality rate of the fish was confirmed by infecting at each concentration with reference to the lethal concentration 50% (LC_50_) confirmed in the previous study [2]. *S. parauberis* PH0710, which was stored at −80 °C, was cultured in BHIA supplemented with 1% NaCl for 24 hours at 27 °C, and a pure colony was isolated and cultured under the same conditions in brain heart infusion broth (BHIB) supplemented with 1% NaCl. After centrifugation at 3000 RPM for 15 min, the supernatant was removed, and the pellet was washed three times with PBS, and centrifuged. The strain was adjusted to 1.5 × 10^4^, 1.5 × 10^5^ and 1.5 × 10^6^ CFU/Fish. Five starry flounders were used per group, and 100 μL of bacteria was subcutaneously injected into each group fish. As a result, all groups of fish died within two weeks, and the experiment was conducted with reference to the concentration in the subsequent infection experiment (Supplementary Fig. 1).

### 2.6 Efficacy by different ceftiofur dose

To determine the efficacy of ceftiofur for each concentration given to starry flounder, the mortality rate of each group was determined by antibiotic concentration after artificial infection. The negative control, positive control, and 20 mg/kg and 40 mg/kg antibiotic treatments were divided into four groups. The body weight of the experimental fish were measured, and the fish were distributed to each experimental water tank. The water temperature was maintained at 24–25 °C. 100 μL of *S. parauberis* PH0710 suspended in PBS was injected subcutaneously in 1.5 × 10^4^ CFU/fish for artificial infection. After 24 hours of bacterial challenge, ceftiofur was diluted in PBS to 20 and 40 mg/kg, and 100 μL was injected into the back muscles of the fish. Fish in the negative control group were injected with PBS instead of bacteria and ceftiofur. The experimental groups were repeated three times. Fish in the positive control group were injected with PBS instead of ceftiofur after a bacterial challenge. Mortality rates were observed for 2 weeks after the injection of antibiotics. During the experiment, bacteria were isolated from kidney and spleen of dead fish and identified by PCR. After 15 days post-challenge, the surviving fish of each group were anaesthetized using benzocaine (Sigma-Aldrich, USA) and dissected, and 0.1 g of kidney was collected. The isolated kidneys were homogenized, suspended in PBS, and plated on BHIA +1% NaCl medium to determine the number of bacteria and the infection rate.

### 2.7 Efficacy by different administration methods

To determine the efficacy of ceftiofur by different administration methods, we examined the mortality rates of each group after artificial infection and antibiotic injection. The fish were divided into intraperitoneal and intramuscular injection groups, and 20 fish were distributed per cage. The water temperature was maintained at 24–25 °C. Then, 100 μL of *S. parauberis* PH0710 suspended in PBS was injected subcutaneously at 5 × 10^3^ CFU/fish for artificial infection. After 24 hours of bacterial challenge, ceftiofur was diluted in PBS to 40 mg/kg, and 100 μL was injected into the fish for each administration methods. The control group was injected with PBS only. Afterwards, the mortality rate was observed for 14 days, and the bacteria were identified. On the 15th day, the infection rate of the surviving fish was confirmed.

### 2.8 Efficacy by different administration methods and temperature

Experiments were carried out at the temperature of 18 °C or 26 °C in three groups of control, intraperitoneal and intramuscular injection groups, with 20 fish per group. Then, 100 μL of *S. parauberis* PH0710 suspended in PBS was injected subcutaneously in 5 × 10^3^ CFU/fish for artificial infection. After 24 hours of bacterial challenge, ceftiofur was diluted in PBS to 40 mg/kg, and 100 μL was injected into the fish for each administration method. The control group was injected with only PBS. Afterwards, the mortality rate was observed for 14 days, and the bacteria were identified. On the 15th day, the infection rate of the surviving fish was confirmed.

### 2.9 Tissue safety test after intramuscular injection

To determine the effect of antibiotics on the host, tissue from the intramuscular injection site was collected, and histopathological examination was performed. Fifteen starry flounder were divided into three groups and maintained at 24–25 °C. The control group was injected with 100 μL of PBS. The other two groups were injected at concentrations of 40 mg/kg and 200 mg/kg ceftiofur. Three fish, one from each group, were anaesthetized by randomly selecting them from each of the groups at 1, 2, 5, 10 and 20 days after injection. The liver, kidney and muscle (antibiotic injection site) were excised and fixed with 10% neutral formalin. Afterwards, each tissue was cut into 4 μm-thick sections, sequentially dehydrated with 70%–100% ethanol, and cleared with xylene and Paraplast, followed by H&E staining. The sections were observed under an optical microscope.

## Appendix A Supplementary data

Supplementary Fig. 1 Determination of cumulative mortality of starry flounder infected with various concentrations of *S. parauberis* PH0710. Supplementary Fig. 1

## References

[1] Mata A.I., Gibello A., Casamayor A., Blanco M.M., Domínguez L., Fernández-Garayzábal J.F. (2004). Multiplex PCR assay for detection of bacterial pathogens associated with warm-water streptococcosis in fish. Appl. Environ. Microbiol..

[2] Cho M.Y., Lee J.I., Kim M.S., Choi H.J., Lee D.C., Kim J.W. (2008). Isolation of *Streptococcus parauberis* from starry flounder, *Platichthys stellatus* Pallas. J. Fish Pathol..

