# Supplementary figures and images for: Efficacy and safety data of ceftiofur antibiotics against Streptococcus parauberis PH0710 infection in starry flounder (Platichthys stellatus)
Source: Data Brief. 2019 Mar 7;23:103729. doi: 10.1016/j.dib.2019.103729 (PMC6660470; doi:10.1016/j.dib.2019.103729)

## Slide 1
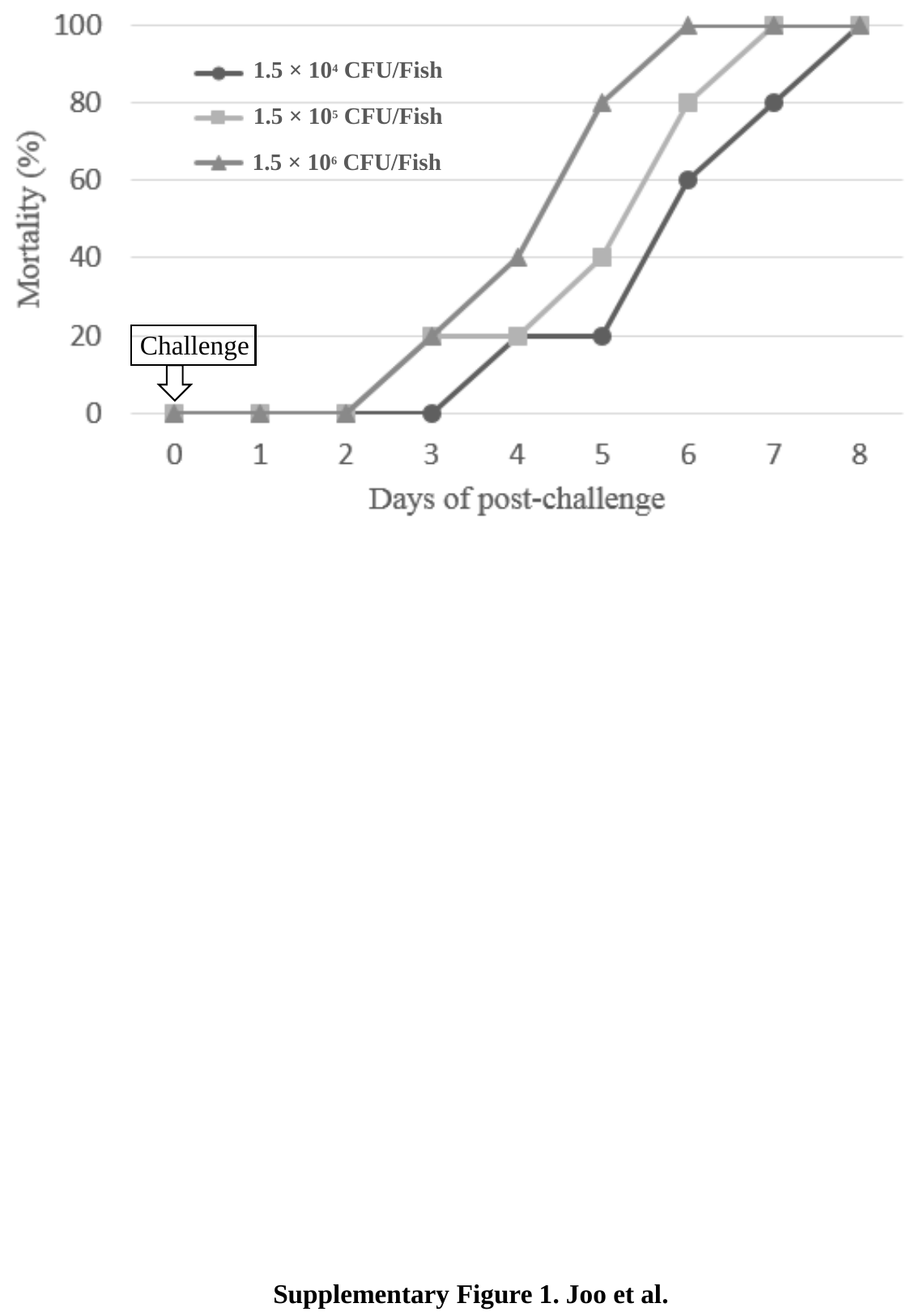

1.5 × 104 CFU/Fish
1.5 × 105 CFU/Fish
1.5 × 106 CFU/Fish
Challenge
Supplementary Figure 1. Joo et al.

Supplement: Supplementary Fig. 1 — Determination of cumulative mortality of starry flounder infected with various concentrations of S. parauberis PH0710. [file mmc2.pptx]
